# Open superior mesenteric artery thrombectomy of dislodged aortic valve causing acute mesenteric ischemia

**DOI:** 10.1016/j.jvscit.2026.102252

**Published:** 2026-04-11

**Authors:** Ivan B. Ye, Alex Houser, Patrick T. Jasinski

**Affiliations:** a Division of Vascular Surgery, Department of Surgery, NYU Long Island School of Medicine, Mineola, NY; b Division of Vascular and Endovascular Surgery, Department of Surgery, Stony Brook Medicine, Stony Brook, NY

**Keywords:** Transcatheter aortic valve replacement, Acute mesenteric ischemia, Embolectomy, Aortic valve, Superior mesenteric artery

## Abstract

Transcatheter aortic valve replacement is preferred for severe aortic stenosis in elderly, high-risk patients. We report the case of a 96-year-old woman who developed acute mesenteric ischemia from embolization of the native heart valve after transcatheter aortic valve replacement and highlight the key management steps that enabled successful revascularization and bowel preservation, as well as the decision-making between surgical and endovascular mesenteric thrombectomy.

Transcatheter aortic valve replacement (TAVR) is the preferred treatment for severe aortic stenosis in elderly patients (≥75 years) and high-risk patients. Compared with open aortic valve replacement, TAVR is generally well-tolerated. Complications include access site bleeding, stroke, paravalvular leakage, acute kidney injury, and conduction abnormalities. After obtaining consent for publication, we present a unique case of a patient who developed acute mesenteric ischemia (AMI) from embolization of native heart valve after TAVR and highlight the key management steps that enabled successful revascularization and bowel preservation and the decision-making between surgical and endovascular mesenteric thrombectomy.

## Case report

A 96-year-old woman with hypertension, type 2 diabetes mellitus, heart failure with preserved ejection fraction, and severe aortic stenosis presented with progressive fatigue, weakness, and worsening dyspnea on exertion. Notably, she lived independently at home. Transthoracic echocardiography revealed a left ventricular ejection fraction of 60%, aortic valve area of 0.66 cm^2^, mean gradient of 23 mm Hg, right atrial pressure of <5 mm Hg, and insufficient tricuspid regurgitation to estimate pulmonary artery systolic pressure. Computed tomography angiography (CTA) demonstrated an annular perimeter of 67.8 mm, annular area of 337.2 mm^2^, right coronary height of 16.0 mm, left coronary height of 12.9 mm, trileaflet valve with severe calcification, mild left ventricular outflow tract calcification, and conventional aortic arch anatomy. Given her age and comorbidities, the patient was scheduled for elective TAVR.

**Fig 1:**
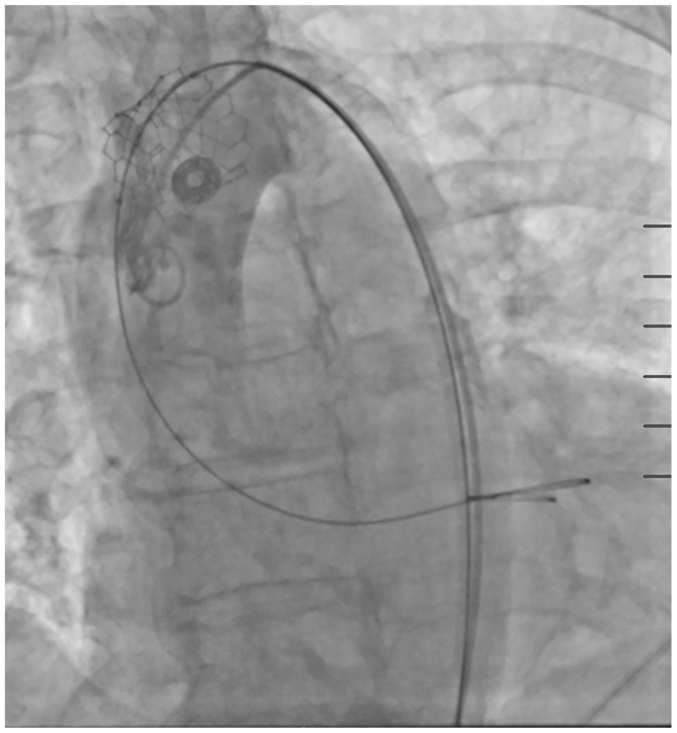
Intraoperative fluoroscopy demonstrating malposition of initial valve in the ascending aorta after deployment.

**Fig 2:**
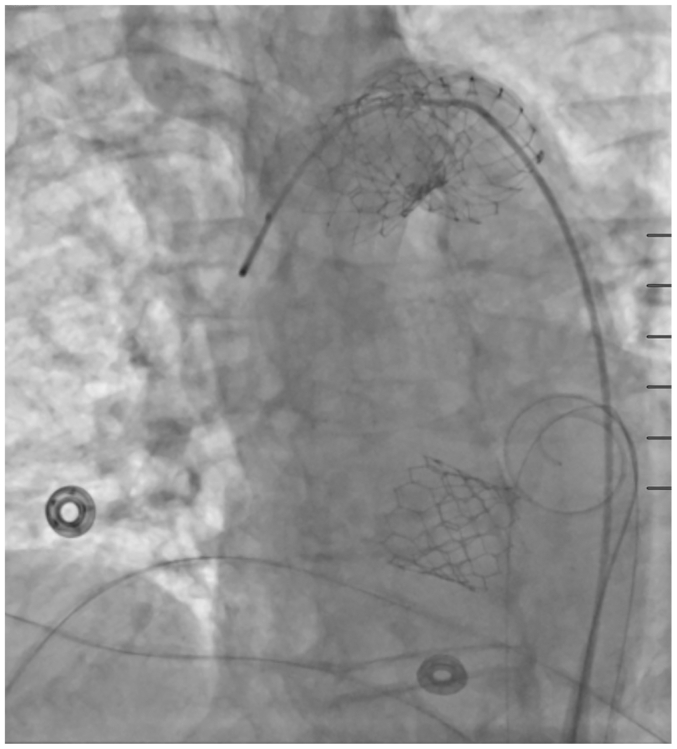
Intraoperative fluoroscopy demonstrating successful deployment of a second valve in the aortic annulus with the first valve stented against the descending thoracic aorta.

During the TAVR procedure, the position and rotation of a 23-mm Edwards valve was confirmed before rapid ventricular pacing. The valve was then deployed using slow controlled balloon inflation. After the balloon was deflated and rapid pacing stopped, the delivery system was removed. On subsequent fluoroscopy, the valve was found to be malpositioned in the ascending aorta (Fig 1). A second valve was then successfully deployed within the native aortic annulus. Completion fluoroscopy demonstrated that the dislodged valve was now in the descending thoracic aorta. A 34-mm Medtronic leafless valve with the valve portion removed was then used as an uncovered stent to tack the malpositioned valve against the aortic wall (Fig 2). Angiography demonstrated patent flow throughout the aorta and its branches, and the procedure was terminated without any access issues.

**Fig 3:**
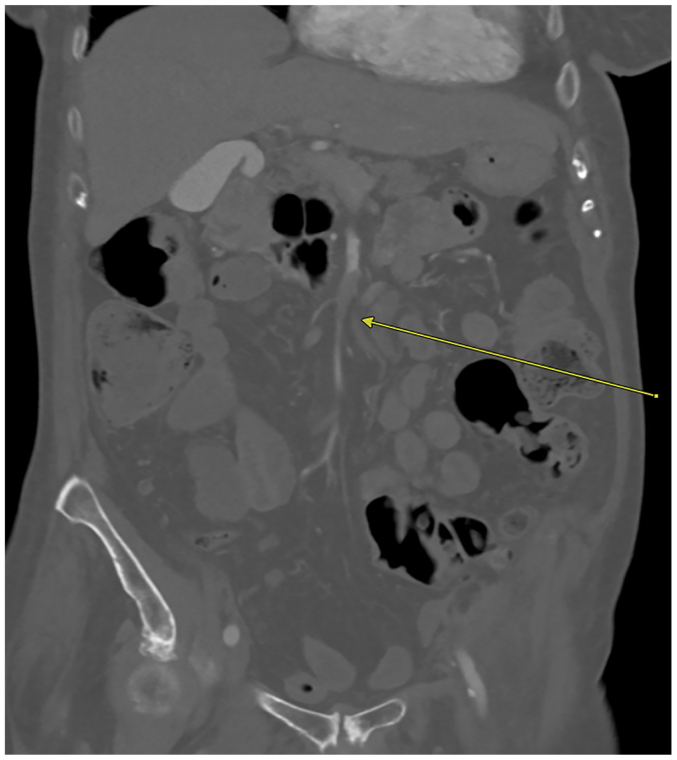
Coronal view of computed tomography angiography (CTA) abdomen/pelvis demonstrating the superior mesenteric artery (SMA) occlusion with distal reconstitution.

**Fig 4:**
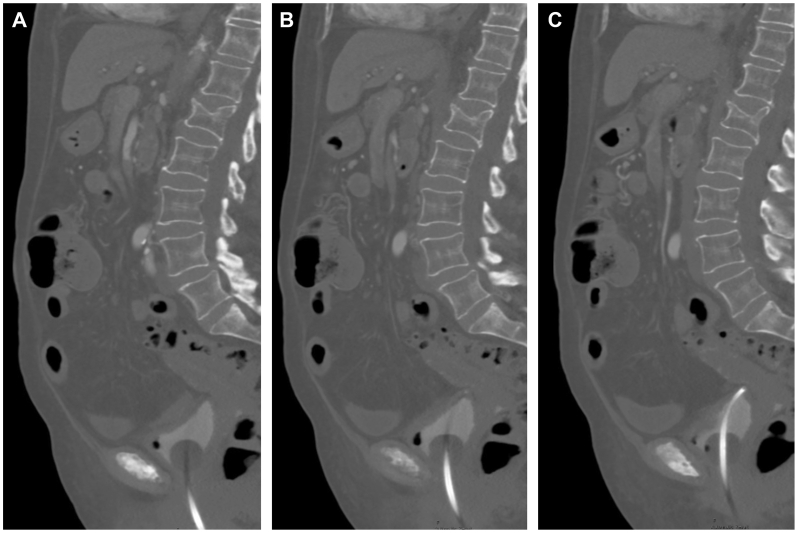
Sagittal views of computed tomography angiography (CTA) of the abdomen/pelvis demonstrating the proximal **(A)**, mid **(B)**, and distal **(C)** extents of the superior mesenteric artery (SMA) occlusion with reconstitution.

On postoperative day 1, the patient developed severe abdominal pain after dinner. She was tachycardic and hypertensive, with new-onset periumbilical tenderness; labs were notable for a severe leukocytosis and lactic acidosis. CTA abdomen/pelvis showed a focal occlusion of the superior mesenteric artery (SMA) trunk with distal reconstitution and edematous, thickened small bowel without pneumatosis or pneumoperitoneum (Figs 3 and 4). After initiation of heparin, she was taken emergently for an exploratory laparotomy and open SMA thrombectomy.

The abdomen was entered via a midline laparotomy. There were patchy areas of dusky bowel without evidence of frank necrosis. After retracting the transverse colon cephalad, we attempted to identify the SMA, which had no pulsatility or Doppler signal in its distal segment. The mesentery was carefully dissected, and the SMA was identified and controlled proximally and distally with silastic vessel loops. A longitudinal arteriotomy was chosen based on surgeon preference because it permits easy proximal or distal extension and was made in a soft, disease-free segment of the SMA. No thrombus or forward bleeding was encountered; however, there was weak back bleeding. A size 3 Fogarty catheter was passed proximally, retrieving a soft tissue mass followed by immediate vigorous pulsatile bleeding.

**Fig 5:**
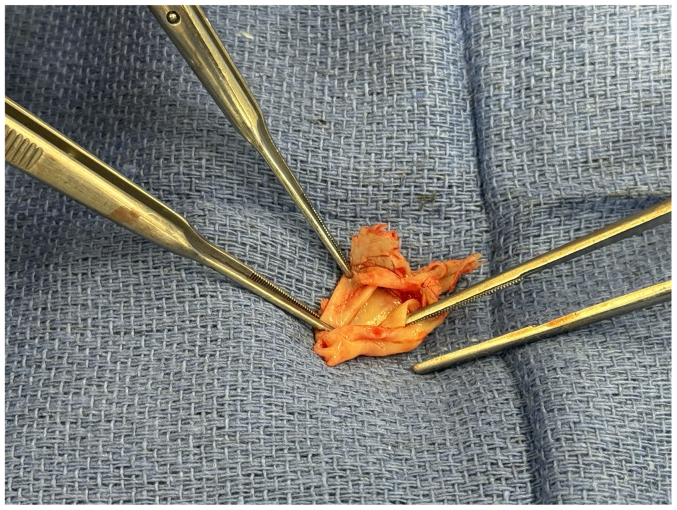
Specimen retrieved during thrombectomy appeared more consistent with native aortic valve tissue than thrombus.

**Fig 6:**
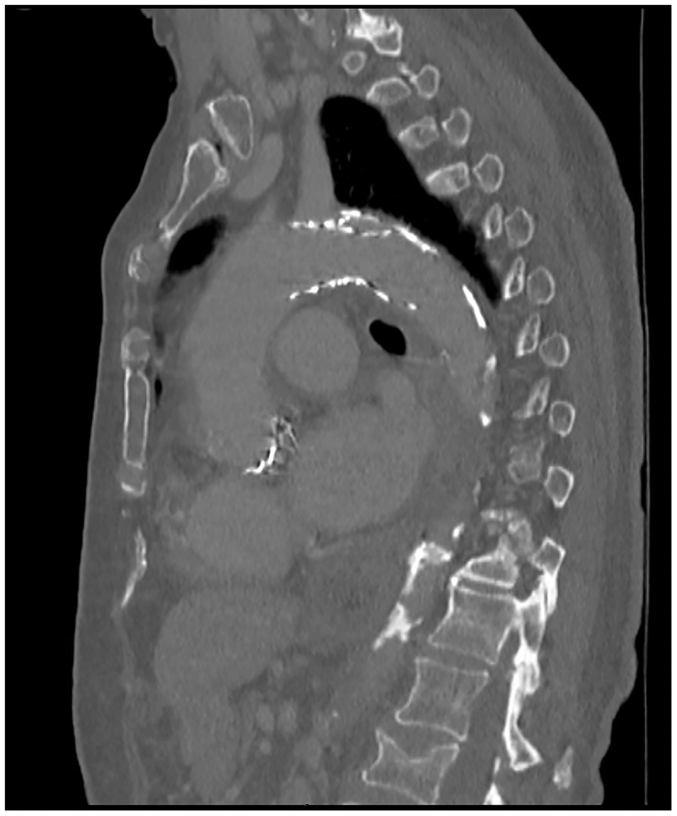
Sagittal view of computed tomography angiography (CTA) of the chest demonstrating intact transcatheter aortic valve replacement (TAVR) and patent aortic stent.

Based on gross intraoperative inspection, the tissue appeared most consistent with native aortic valve tissue rather than thrombus (Fig 5). The specimen was submitted for pathology but was subsequently lost, precluding histopathological analysis. A patch angioplasty was performed with bovine pericardium and, upon completion, a pulse was appreciated along the SMA. The small bowel showed improved perfusion shortly thereafter; she was left open and closed 24 hours later. No bowel resection was required. She recovered uneventfully and was discharged on postoperative day 7 on aspirin 81 mg/d. The patient was seen in vascular and general surgery clinic 2 weeks later with a healed midline incision, tolerating diet, and repeat CTA of the abdomen/pelvis demonstrating a patent aortic stent and SMA (Fig 6). The patient continues to do well 6 months after surgery.

## Discussion

AMI is a rare but devastating complication after TAVR with high morbidity and mortality. Typical causes include embolism, thrombosis of calcified mesenteric vessels, and nonocclusive mesenteric ischemia. We describe a unique mechanism of AMI after TAVR and highlight management principles that contributed to bowel preservation and a favorable outcome.

Embolic AMI after TAVR may arise from atrial thrombus, aortic atheroma, or thrombus at prior aortic prosthetic grafts. Embolization can occur during wire and device manipulation across the aortic arch, valve deployment, and balloon postdilation. The overall incidence of post-TAVR thromboembolic, including stroke, is approximately 4%.^1^ Yamashita et al^2^ reported observing dislodgement of a protruding plaque on transesophageal echocardiography during device advancement through the descending aorta. Selective angiography demonstrated an SMA occlusion and the plaque was successfully aspirated endovascularly. In a prospective cohort of 269 TAVR patients, Chen et al^3^ reported a 30-day AMI incidence of 1.5% with all four affected patients dying within 2 weeks of TAVR.

Acute thrombosis of the SMA is a rare cause of AMI after TAVR. Del Val et al^4^ reported rupture of a calcified plaque at SMA origin after TAVR, resulting in complete SMA occlusion and ischemia of the ascending and transverse colon; despite emergent bowel resection, the patient developed multiorgan failure and died 3 days post TAVR.

TAVR-associated splanchnic vasoconstriction can reduce SMA flow and precipitate nonocclusive mesenteric ischemia, which has been reported in several cases.^5^, ^6^, ^7^, ^8^ Noguchi et al^5^ documented SMA vasospasm after TAVR on selective angiography, which was successfully treated with continuous intra-arterial papaverine infusion. Rapid pacing during valve deployment may further depress cardiac output and exacerbate mesenteric hypoperfusion, particularly in patients with severe SMA stenosis. Chen et al^3^ found that >70% SMA stenosis was an independent risk factor for developing AMI after TAVR and was associated with higher 1-year all-cause mortality (adjusted hazard ratio, 3.78; 95% confidence interval; 1.74-8.19; *P* = .001). Accordingly, some European guidelines recommend treating chronic mesenteric ischemia before TAVR.^9^ However, the optimal management of asymptomatic SMA stenosis before TAVR remains unclear.

In this case, the first deployed valve dislodged into the descending aorta, which was then secured by an uncovered stent. During this manipulation, the native aortic valve likely sheared off and embolized into the proximal SMA trunk. The patient likely developed symptoms 1 day later when the aortic valve embolized distally and caused a focal occlusion of the distal SMA. After the CTA findings, a multidisciplinary discussion was held between vascular surgery, general surgery, and cardiology (primary team). At our institution, in the absence of any signs and symptoms of bowel ischemia, endovascular SMA thrombectomy is typically the first option, particularly for this elderly patient with high-risk comorbidities. However, given the patient's acute abdominal pain, leukocytosis, lactic acidosis, and small bowel edema on CT scan, general surgery felt that the integrity of the bowel required exploration. Thus, exploratory laparotomy was chosen to evaluate the bowel while also allowing for open SMA thrombectomy.

In retrospect, an endovascular approach would likely have been unsuccessful for the retrieval of the embolized aortic valve. Manual aspiration and aspiration-based thrombectomy systems are unlikely to capture dense valvular tissue within the catheter lumen in contrast with soft thrombus. Rheolytic and rotational thrombectomy systems can debulk large thrombus, but their efficacy against valvular tissue is uncertain. Finally, thrombolysis would be ineffective, and stenting a bulky noncompressible valve may result in inadequate stent expansion and persistent obstruction. Additional time spent attempting endovascular thrombectomy may prolong ischemic time. Avulsion of the native aortic valve leaflet during TAVR has been scarcely reported in the literature.^10^, ^11^, ^12^, ^13^ In cases of partial avulsion, observation and percutaneous snare retrieval were successful in patients without impacted hemodynamics.^10^^,^^11^ In other case reports, patients underwent surgical resection and explant of the avulsed valve before proceeding with open aortic valve repair.^12^^,^^13^ To our knowledge, this is the first case report of a complete avulsion of the aortic valve during TAVR that embolized and caused AMI.

This patient made a full recovery from post-TAVR AMI, highlighting the importance of having a high index of suspicion for AMI. Expedited axial imaging was obtained to localize the occlusion, assess bowel perfusion, confirm valve and stent position, and exclude dissection. Multidisciplinary effort is essential in consulting and coordinating with general surgery and vascular surgery for operative intervention. Ultimately, the patient was taken to the operating room within 2 hours of symptom onset, where early successful revascularization preserved bowel viability.

## Funding

None.

## Disclosures

None.
